# *Mammea siamensis* Flower Extract-Induced Cell Death Apoptosis in HCT116 Colon Cancer Cells via Vacuolar-Type H^+^-ATPase Inhibition Associated with GSK-3β/β-Catenin, PI3K/Akt/NF-κB, and MAPK Signaling Pathway

**DOI:** 10.3390/ph18040441

**Published:** 2025-03-21

**Authors:** Pornnapa Sitthisuk, Watcharaporn Poorahong, Sukanda Innajak, Aungkana Krajarng, Siritron Samosorn, Ramida Watanapokasin

**Affiliations:** 1 Department of Biochemistry, Faculty of Medicine, Srinakharinwirot University, Bangkok 10110, Thailand; pornnapa.sitthisuk@g.swu.ac.th (P.S.); suinnajak@gmail.com (S.I.); 2 Department of Biochemistry, Faculty of Medicine, Bangkok Thonburi University, Bangkok 10170, Thailand; noi.poorahong@gmail.com; 3 Chulabhorn International College of Medicine, Thammasat University, Pathum Thani 12120, Thailand; krajarng@tu.ac.th; 4 Department of Chemistry and Center of Excellence for Innovation in Chemistry, Faculty of Science, Srinakharinwirot University, Bangkok 10110, Thailand; siritron@g.swu.ac.th

**Keywords:** *Mammea siamensis*, apoptosis, colon cancer, MAPK, PI3K/Akt/NF-κB, GSK-3β/β-catenin, V-ATPases

## Abstract

**Background and Objective:** *Mammea siamensis* (MS) is a Thai herb used in traditional medicine. Previous studies have reported the antiproliferative effects of its constituents in various cancer cell lines. However, the effects of MS extract on cytotoxicity and molecular mechanisms of apoptosis induction in HCT116 colon cancer cells have not been fully explored. **Methods and Results:** The cytotoxic effect of MS extract on HCT116 cells was assessed using the MTT assay. MS extract increased cytotoxicity in a concentration-dependent manner. It also induced nuclear morphological changes and disrupted the mitochondrial membrane potential (*ΔΨ*m), as assessed by Hoechst 33342 and JC-1 staining, respectively. These findings indicated that MS extract induced apoptosis, which was further confirmed by flow cytometry showing an increase in the sub-G1 phase. To investigate the expression of signaling proteins, Western blot analysis was conducted. The results showed that MS extract activated caspase activity (caspase-8, -9, and -7) and inhibited PARP activity. Additionally, MS extract upregulated pro-apoptotic proteins (tBid, Bak, and cytochrome c) while downregulating anti-apoptotic proteins (Bcl-2 and Bcl-xL). Mechanistic studies revealed that MS extract activated MAPK pathways while inactivating the PI3K/Akt/NF-κB and GSK-3β/β-catenin pathways. Notably, MS extract also inhibited V-ATPases, as evaluated by acridine orange staining and Western blot analysis. **Conclusions:** Our findings suggest that MS extract induces apoptosis via the activation of both intrinsic and extrinsic pathways associated with the key signaling pathways. Therefore, MS extract shows potential as a therapeutic agent for colon cancer.

## 1. Introduction

Colon cancer (CRC) is the second leading cause of cancer-related mortality and the third most common type of cancer globally. Recent statistics indicate that approximately 1.9 million people were diagnosed with CRC in 2020 [1]. Most diagnoses occur in individuals over the age of 50, making CRC a significant public health concern in many countries worldwide [2]. Various cancer treatments, including surgery, chemotherapy, targeted therapy, immunotherapy, and radiation, are available. However, these treatments are often prohibitively expensive for low-income patients and can also cause side effects to healthy cells in the body [3,4]. Therefore, there is an urgent need to search for new agents for cancer treatment.

*Mammea siamensis* (Miq.) T. Anders, known locally in Thai as Sarapi or Saraphi, belongs to the Calophyllaceae family. *M. siamensis* is a Thai herb used in traditional medicine, such as a heart tonic, to reduce fever and to increase appetite [5]. Previous chemical studies of the flowers of *M. siamensis* have reported the isolation of various bioactive compounds, including several coumarins, xanthone, flavonoids, and other such bioactive compounds [5,6]. This plant and its constituents have demonstrated various biological activities. Compounds isolated from the flowers of *M. siamensis* have exhibited antimalarial potential, showing inhibitory effects against *Plasmodium falciparum*, the parasite responsible for malaria. Using the pLDH assay, these compounds displayed IC_50_ values ranging from 9.57 to 29.32 µM [7]. In addition, an in vivo study was conducted to evaluate the antimalarial potential of 1-hydroxy-5,6,7-trimethoxyxanthone (HTX), a compound isolated from *M. siamensis* flowers. At a dosage of 10 mg/kg body weight, HTX significantly suppressed parasitemia in malaria-infected mice by 74.26%, demonstrating strong antimalarial efficacy. Furthermore, no acute toxicity or significant adverse effects were observed at doses up to 50 mg/kg, suggesting a favorable safety profile [8]. *M. siamensis* extract has also demonstrated notable antioxidant activity, with an EC_50_ value of 10.17 µg/mL in the DPPH radical scavenging assay and an IC_50_ value of 0.43 µg/mL in the lipid peroxidation assay [9]. Additionally, the extract exhibited strong antimicrobial activity, showing the highest efficacy against *Staphylococcus aureus*, methicillin-resistant *Staphylococcus aureus* (MRSA), and *Staphylococcus epidermidis*, with minimum inhibitory concentration (MIC) values of 0.005, 0.005, and 0.039 mg/mL, respectively, and minimum microbicidal concentration (MMC) values matching these MIC values [10].

Interestingly, *M. siamensis* and its components have demonstrated anti-proliferative effects against various cancer cell lines. The MTT assay results revealed that several coumarins exhibited IC_50_ values ranging from 1.1 to 95.0 µM in human digestive tract carcinoma cells (HSC-2, HSC-4, and MKN-45) and human breast adenocarcinoma (MCF-7) [5]. Additionally, the methanol extract of *M. siamensis* flowers and its fractions showed potent anti-proliferative activity against human prostate carcinoma cells (LNCaPs), as determined by the Cell Counting Kit-8 (CCK-8) assay. The IC_50_ values for the extract and its fractions ranged from 2.0 to 23.8 µg/mL, while the isolated coumarin constituents exhibited IC_50_ values between 0.12 and 61.9 µM [6]. Moreover, in colon cancer cells (HCT116), the XTT assay demonstrated that Mammea A/AA and the steroid MSHE1, both isolated from *M. siamensis*, had IC_50_ values of 6.6 µM and 6.5 µM, respectively [11]. Kayeassamin A was isolated from the *M. siamensis* flower and has been shown to have anti-cancer activity in human leukemia HL-60 cells via apoptosis induction [12]. Interestingly, *M. siamensis* is utilized both in Ayurveda and by indigenous communities in Northeast Thailand for cancer prevention and healing purposes [11]. The “Poh-Pu” remedy, a Traditional Thai Medicine (TTM) formulation used in cancer treatment, includes *M. siamensis* as one of its components. This remedy has been prescribed for over thirty years at the Jitmeatta Mercy Foundation for Cancer Patients (JFCT) in Phetchaburi province, Thailand [11,13], which indicates that this plant has shown potential for anti-cancer effects. However, previous studies have primarily focused on the anti-proliferative effects of *M. siamensis* extract in HCT116 colon cancer cells, while the molecular mechanisms underlying its action remain unexplained.

Recently, apoptosis inducers have been utilized in cancer treatment to help control or potentially halt the uncontrolled growth of cancer cells, aiming to inhibit their development and progression [14,15,16]. Apoptosis is a programmed cell death that follows two primary pathways: the extrinsic and intrinsic pathways. The intrinsic, or mitochondrial pathway, is triggered by internal signals, such as DNA damage, ischemia, and oxidative stress, while external signals activate the extrinsic pathway. The intrinsic apoptosis pathway is started with cytochrome c releasing from mitochondria, and, then, caspase-9 is activated, which subsequently activates caspases-3 or -7, leading to cell death. Moreover, the Bcl-2 family proteins also play a key role in the intrinsic pathway. Conversely, the extrinsic pathway is associated with ligand binding to death receptors on the cell surface, resulting in the activation of caspase-8. Activated caspase-8 activates caspases-3 or -7 then cleaves Bid (pro-apoptotic protein in the Bcl-2 family) into the truncated form of Bid (tBid). Then, tBid promotes the release of cytochrome c from mitochondria [16,17,18]. The abnormal apoptosis is associated with a variety of diseases, including cancer. Dysregulated apoptosis results in the accumulation of damaged cells, ultimately leading to tumorigenesis, as well as resistance to cancer therapy. Additionally, interference with cell signaling pathways, including the glycogen synthase kinase-3β (GSK-3β)/β-catenin, phosphoinositide 3-kinase (PI3K)/protein kinase B (PKB, Akt), and mitogen-activated protein kinase (MAPK) pathway, plays an important role in cancer development and progression [19,20,21].

Vacuolar ATPase (V-ATPase) is an ATP-driven H⁺ pump that transports protons across both intracellular and plasma membranes, regulating the pH levels inside and outside of cells. This regulation is essential for various cellular functions, including cancer cell growth, invasion, metastasis, and proliferation [22]. Recently, V-ATPases have emerged as a promising target for cancer therapy [23]. Research has shown that V-ATPases are overexpressed in various cancers, where they play a role in promoting cancer cell proliferation and metastasis [24,25]. Notably, inhibiting V-ATPase activity has been found to trigger apoptosis [23], suggesting that targeting V-ATPases could be a valuable strategy for developing new anti-cancer treatments.

Therefore, this study explored the impact of MS extract on cytotoxicity and apoptosis induction, as well as the molecular mechanisms involved in HCT116 human colon cancer cells. The findings provide new insights into the potential of MS extract as an anti-cancer agent for colon cancer and its possible therapeutic applications and its possible use in synergy with cancer treatments in the future.

## 2. Results

### 2.1. ^1^H NMR Analysis of MS Methanolic Extract

**Figure 1:**
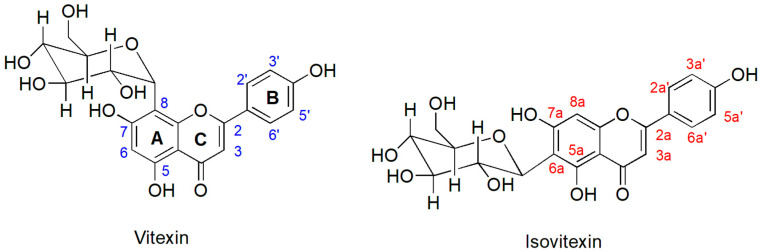
Chemical structures of vitexin and isovitexin, showing ring names (**A**, **B**, **C**) and position numbering (a and a′ indicating isovitexin positions).

**Figure 2:**
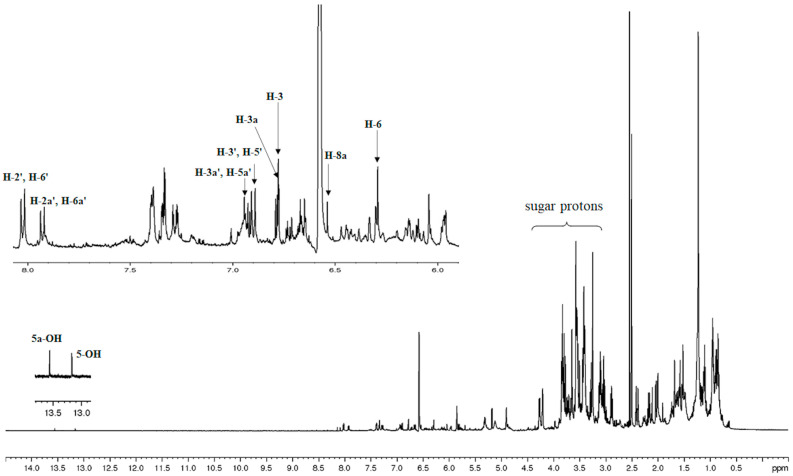
^1^H-NMR spectrum with expansion of MS methanolic extract in DMSO-*d6*.

^1^H NMR spectroscopic analysis of the MS methanolic extract revealed that the extract contained two *C*-glycosyl flavones, vitexin and isovitexin, as shown in Figure 1. The structure of these compounds in the extract was elucidated by analysis of their 1D and 2D ^1^H NMR spectroscopic data and by comparison with the literature values (Figure 2) [26,27]. The presence of two singlets at 13.17 and 13.56 ppm, characteristic signals of the 5-OH proton of the flavonoid skeleton, were observed. Two singlets at 6.29 and 6.54 were ascribed to H-6 and H-8a on ring A. Four doublets with *ortho*-coupling (*J* = 10 Hz) at 6.90, 6.93, 7.93, and 8.02 ppm were attributed to H-3′ and H-5′, H-3a′ and H-5a′, H-2a′ and H-6a′, and H-2′ and H6′, respectively, on aromatic ring B, which are typical patterns of the asymmetrical *p*-disubstituted benzene ring. In addition, from the gCOSY experiment, the signal at 6.90 was correlated to the signal at 8.02, and the signal at 6.93 was correlated to the signal at 7.93 ppm. Two singlets at 6.77 and 6.78 ppm were ascribed to H-3 and H-3a on ring C. Therefore, it could be concluded that the NMR spectral data corresponded to those previously reported for the mixture of vitexin and isovitexin detected in *M. siamensis* T. Anders [27].

### 2.2. MS Extract Increased Cell Toxicity in HCT116 Cells

**Figure 3:**
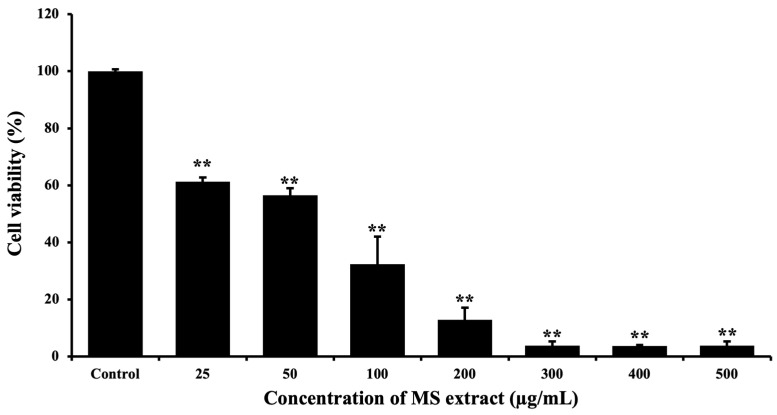
Effects of MS extracts on cytotoxicity in HCT116 cells. Cells are treated with various concentrations of MS extract for 24 h and examined by using the MTT assay. The percentage of cell viability is shown as mean values ± SD of three independent experiments. ** *p* < 0.01 suggest a statistically significant difference relative to the control.

The MTT assay was used to evaluate cytotoxicity in HCT116 cells. We treated the cells with various concentrations of MS extract for 24 h. The viability of the cells in response to the treatment decreased in a dose-dependent manner compared with the untreated control, with an IC_50_ of 79.57 ± 0.25 µg/mL (Figure 3), indicating increased cell toxicity. Meanwhile, an IC_50_ of 301.3 ± 1.27 µg/mL was observed in normal Vero cells (Figure S1). This result suggests the potential of MS extract on cell proliferation inhibition in HCT116 cells.

### 2.3. MS Extract Induced Nuclear Morphological Change in HCT116 Cells

**Figure 4:**
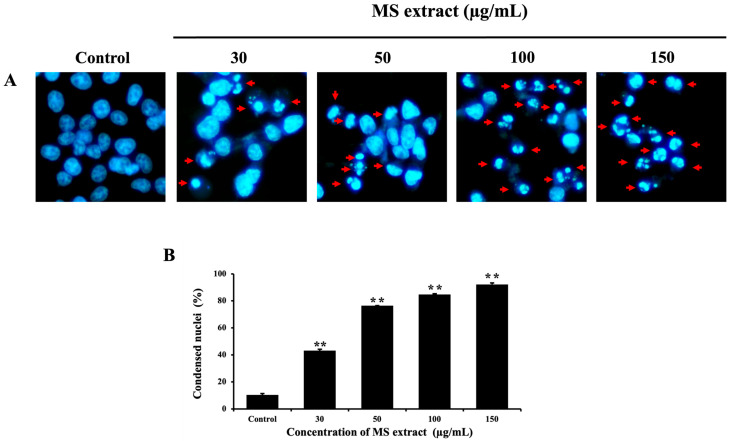
Effects of MS extracts on nuclear morphological change in HCT116 cells: (**A**) Fluorescence pictures of Hoechst 33342 staining captured with a fluorescence microscope (20×). Red arrows indicate condensed HCT116 cells and the presence of apoptotic bodies. (**B**) Histogram showing the proportion of nuclear-condensed cells compared to control cells. ** *p* < 0.01 suggest a statistically significant difference relative to the control.

Nuclear condensation is a characteristic of nuclear morphological changes in apoptotic cells [17,28], which can be evaluated by Hoechst 33342 staining and visualized under a fluorescence microscope. As illustrated in Figure 4, the number of condensed HCT116 cells and apoptotic bodies (indicated by red arrows) increased with higher doses of MS extract. This suggests that MS extract-induced apoptosis may play a role in the decreased viability of HCT116 cells.

### 2.4. MS Extract Reduced Mitochondrial Membrane Potential (ΔΨm) in HCT116 Cells

**Figure 5:**
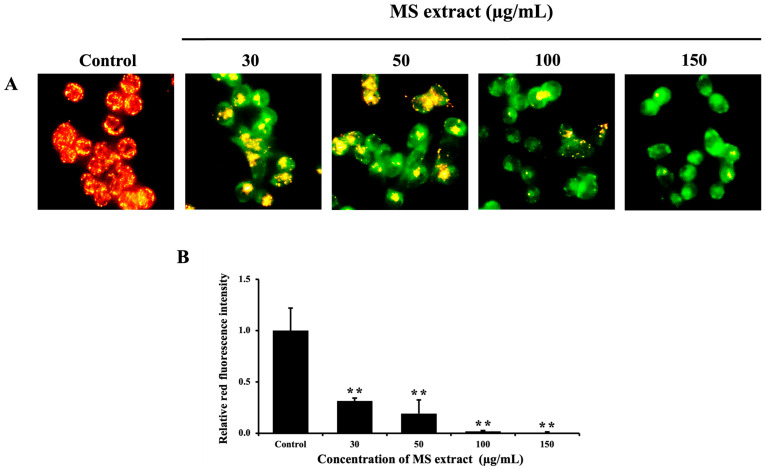
Effects of MS extracts on *ΔΨ*m in HCT116 cells: (**A**) Fluorescence pictures of JC-1 staining captured with a fluorescence microscope (20×). An increase in green fluorescence signifies a reduction in *ΔΨ*m. (**B**) Histogram showing the relative red fluorescence intensity compared to control cells. ** *p* < 0.01 suggest a statistically significant difference relative to the control.

Mitochondrial membrane potential plays a critical role in regulating intrinsic apoptosis, as investigated using JC-1 staining [29]. The findings showed a significant reduction in the red fluorescence intensity of JC-1 aggregates in the treated cells compared to the control group, indicating a decrease in *ΔΨ*m (Figure 5). This suggests that the MS extract may induce apoptosis in HCT116 cells via induced mitochondrial dysfunction.

### 2.5. MS Extract Increased the Population of Sub-G1 Apoptotic HCT116 Cells

**Figure 6:**
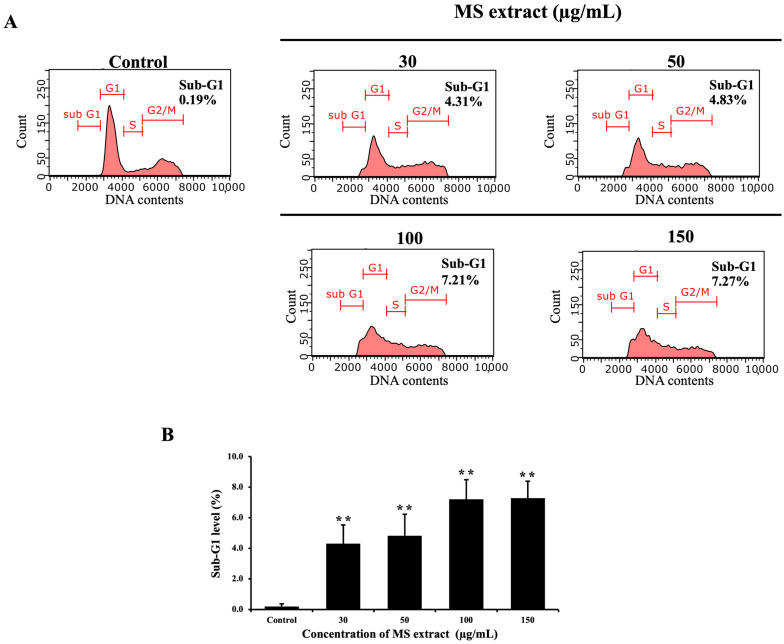
Effects of MS extracts on the sub-G1 population in HCT116 cells: (**A**) Representative histograms analyzed by flow cytometry. (**B**) Percentage of cells in the sub-G1 phase relative to control. ** *p* < 0.01 suggest a statistically significant difference relative to the control.

Apoptotic cells are identified by their decreased DNA content, which can be measured using flow cytometry through the sub-G1 DNA content [28]. Therefore, HCT116-treated cells were stained with Guava Cell Cycle^®^ reagent, allowing us to measure DNA content by flow cytometry. After 24 h of treatment with MS extract, a significant increase in the percentage of the sub-G1 population was observed, correlating with increased dose levels (Figure 6).

### 2.6. MS Extract Stimulated Apoptosis Pathways in HCT116 Cells

**Figure 7:**
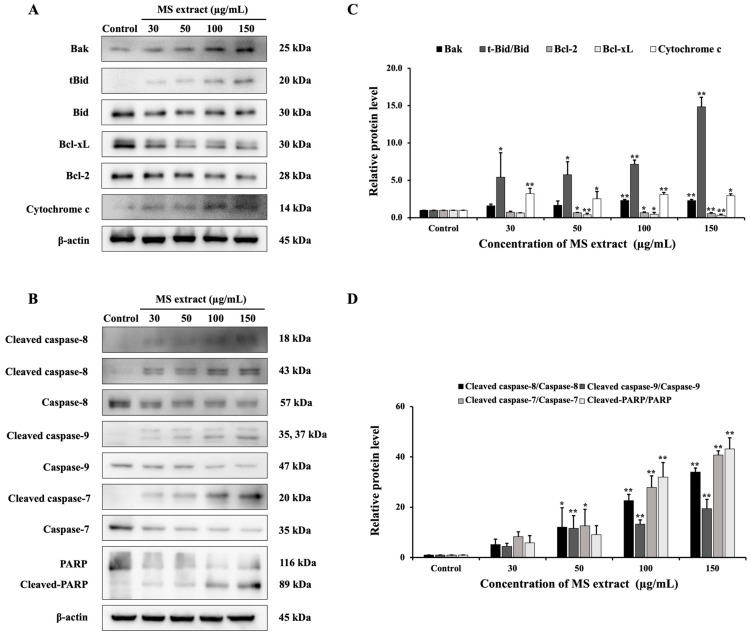
The effects of MS extracts on the protein expression of (**A**) Bcl-2 family proteins, (**B**) caspase, and PARP in HCT116 cells are detected by Western blot analysis. (**C**,**D**) The intensity of the bands in relation to the control. * *p* < 0.05 and ** *p* < 0.01 suggest a statistically significant difference relative to the control.

The molecular mechanisms of apoptosis induction are triggered through two main pathways: the extrinsic and intrinsic pathways [17]. Therefore, we investigated protein expression in these pathways to elucidate the molecular mechanisms of the MS extract. As depicted in Figure 7, Western blot analysis revealed that the expression of the anti-apoptotic protein Bcl-2 decreased in comparison to the pro-apoptotic Bax protein, as well as cytochrome c, which was enhanced. The expression of cleaved caspase-9 and caspase-7, as well as cleaved PARP, also increased following MS extract treatment. These results suggest the activation of intrinsic pathways. Additionally, the MS extract increased the levels of cleaved caspase-8, indicating that the extrinsic pathways are involved. This resulted in the increased truncation of Bid (tBid) in the MS-treated cells, suggesting that there is a cross-talk between the extrinsic and intrinsic pathways.

### 2.7. MS Extract Inhibited V-ATPase Activity in HCT116 Cells

**Figure 8:**
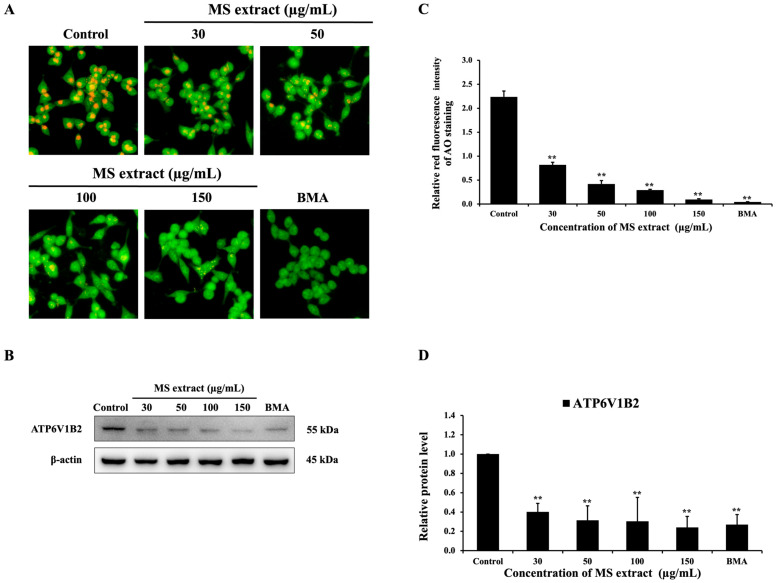
Effects of MS extracts on V-ATPase activity in HCT116 cells: (**A**) Fluorescence microscopy images of AO-stained cells (20 X). The orange or red fluorescence indicates acidic vesicular organelles (AVOs), which might be generated by V-ATPases. BMA is used as a positive control. (**B**) The protein expression of ATP6V1B2 is detected by Western blot analysis. (**C**) The histogram represents the relative red fluorescence intensity of AO staining compared to the control. (**D**) The intensity of the bands in relation to the control. ** *p* < 0.01 suggest a statistically significant difference relative to the control.

Previous studies have reported that inhibitors of V-ATPases can induce apoptosis [30]. In our current study, MS extract shows the potential to induce apoptosis. Thus, we employed AO staining and Western blot to explore whether MS extract could inhibit V-ATPase activity in HCT116 cells. The results showed that MS extract can inhibit V-ATPase activity by suppressing the orange or red fluorescence of AO dye compared to the control group (Figure 8A). The results of the Western blot showed that the protein level of the B2 subunit of H^+^-ATPase (ATP6V1B2) was decreased (Figure 8B). These results are consistent with those obtained using Bafilomycin A1 (BMA), a specific V-ATPase inhibitor. Therefore, these findings demonstrate that MS extract is a potent V-ATPase inhibitor for the treatment of HCT116 cells.

### 2.8. MS Extract Relegated the GSK-3β/β-Catenin, PI3K/Akt/NF-κB, and MAPK Signaling Pathways in HCT116 Cells

**Figure 9:**
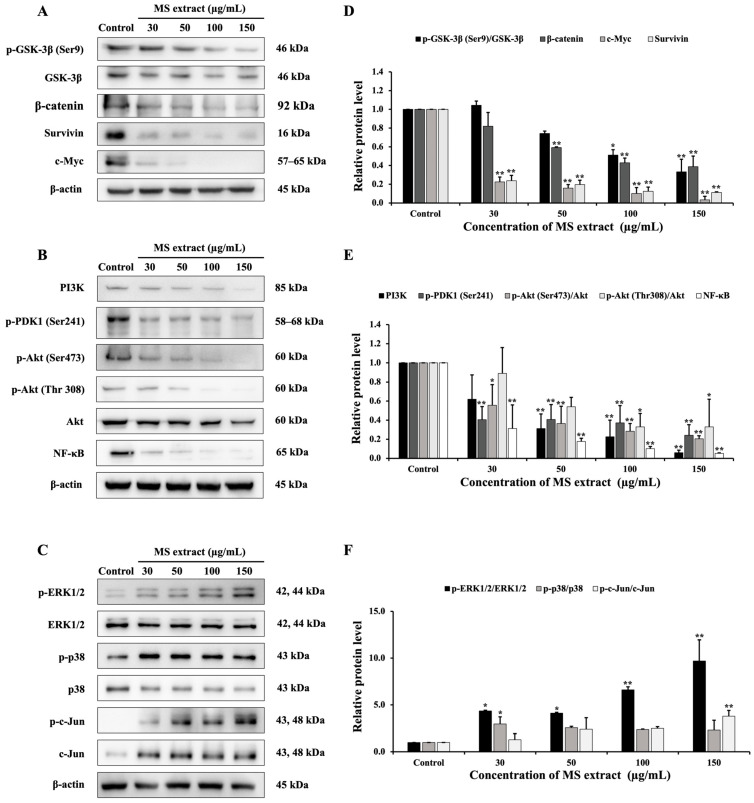
Effects of MS extract on protein expression involving (**A**) the GSK-3β/β-catenin pathway, (**B**) the PI3K/Akt/NF-κB pathway, and (**C**) the MAPK pathway in HCT116 cells, which are detected by Western blot analysis. (**D**–**F**) The intensity of the bands in relation to the control. * *p* < 0.05 and ** *p* < 0.01 suggest a statistically significant difference relative to the control.

There are many cellular signaling pathways involved in cancer. Therefore, a Western blot assay was performed to examine the expression of proteins related to the GSK-3β/β-catenin, PI3K/Akt, and MAPK signaling pathways in order to further investigate the mechanisms of the MS extract in HCT116 cells. First, results showed that MS extract downregulated p-GSK-3β, β-catenin, c-Myc, and survivin (Figure 9A). Next, the expressions of PI3K, p-PDK1, p-Akt (Ser473), p-Akt (Thr308), and NF-κB were also downregulated (Figure 9B). Finally, MS extract upregulated the phosphorylated forms of ERK (p-ERK), p38 (p-p38), and c-Jun (p-c-Jun), as shown in Figure 9C.

## 3. Discussion

Herbal plants have been traditionally used in folk medicine across many countries worldwide. Natural extracts from the fruits, seeds, leaves, bark, flowers, and roots of medicinal plants offer numerous benefits, including skin care, food enhancement, and nutritional improvement [31]. *Mammea siamensis* is a medicinal plant with a long-standing history, particularly in Thailand. In traditional Thai medicine, the flowers are valued for their cardiotonic properties and are used to reduce fever and stimulate appetite [32]. Interestingly, *M. siamensis* is one ingredient in the “Poh-Pu” remedy, a traditional Thai medicine (TTM) formula that has been prescribed for over 30 years at the Jitmeatta Mercy Foundation for Cancer Patients (JFCT) in Phetchaburi province. A previous study investigating this remedy found that cancer patients held a positive attitude toward TTM, exhibited strong confidence in clinical practitioners, and maintained their quality of life. This research employed both quantitative and qualitative methods, including structured questionnaires and in-depth interviews, with data collected from 408 patients [11,13,33]. Despite its extensive applications in traditional medicine, research on *M. siamensis* in cancer treatment remains limited. Modern scientific studies have reported that the ethanolic extract of *M. siamensis* showed the highest activity against HepG2 cells, with an IC_50_ value of 3.15 ± 0.16 μg/mL, among the plant components in the TTM remedy [13].

These findings highlight growing interest in exploring the potential of *M. siamensis* for cancer therapy. However, further research is necessary to scientifically validate the traditional uses of *M. siamensis* flowers. Additionally, understanding their mechanism of action is crucial for developing effective, natural-based treatments. Therefore, this study aims to investigate the molecular mechanism of *M. siamensis* extract in HCT116 colon cancer cells, as existing data remain unclear. Several previous studies indicate that *M. siamensis* and its constituents exhibit anti-cancer properties by inhibiting cell proliferation across various cancer cell types. A methanol extract from the flowers of *M. siamensis* demonstrated significant anti-proliferative effects against human prostate carcinoma LNCaP cells, with an IC_50_ value of 2.0 µg/mL. Additionally, specific coumarin compounds, including mammeasins A (3, IC_50_ = 1.2 µM) and B (4, IC_50_ = 0.63 µM), sugangin B (18, IC_50_ = 1.5 µM), kayeassamins E (24, IC_50_ = 3.0 µM), and G (26, IC_50_ = 3.5 µM), as well as mammeas E/BA (40, IC_50_ = 0.88 µM), E/BB (41, IC_50_ = 0.52 µM), and E/BC (42, IC_50_ = 0.12 µM), exhibited relatively strong anti-proliferative activity [6]. In the human cancer cell lines MDA-MB-231 (breast cancer), HCT-116 (colon cancer), and CCRF-CEM (leukemia) have all been significantly affected by the cytotoxic activity of coumarins such as mammea A/AA, with IC_50_ values of 7.2, 6.6, and 20.9 µM, respectively, which are extracted from *M. siamensis* flowers [11]. Our findings align with these reports, demonstrating that MS extract significantly decreases cell proliferation in HCT116 cells.

While prenylated coumarins are prominent, the plant also contains significant flavonoid compounds. Previous investigations have reported that *M. siamensis* contains high levels of vitexin and isovitexin, well-known bioactive flavonoid compounds recognized for their anti-cancer and anti-tumor properties [27,34]. In our study, we conducted a chemical analysis of *M. siamensis* flowers using ^1^H NMR. As shown in Figure 1, we identified vitexin and isovitexin in the methanolic extract, which may contribute to the cytotoxic effects observed in HCT116 cells. Overall, these studies suggest that the chemical constituents of *M. siamensis* play a key role in inhibiting cancer cell proliferation.

Flavones are a group of flavonoids named after their common yellow color, derived from the word flavus. They are based on the backbone of 2-phenylchromen-4-one (2-phenyl-1-benzopyran-4-one) (flavone). Vitexin and isovitexin belong to the class of flavones and are derivatives of apigenin with 8/6-C-glucoside. Their chemical properties are very similar. Vitexin, also known as 8-d-glucosyl-4′,5,7-trihydroxy-flavone, or apigenin-8-C-glucoside, with molecular formula C_21_H_20_O_10_, and molecular weight 432.3775 g/mol. In contrast, isovitexin, an isomer of vitexin, contains a 6-C-glucosyl [35]. Vitexin and isovitexin exhibit a range of pharmacological effects, including anti-cancer properties. Previous studies indicated that vitexin and isovitexin contribute to the inhibition of cancer cell proliferation through various mechanisms, including apoptosis induction [36]. Vitexin, found in the traditional Chinese herb Crataegus pinnatifida (hawthorn), has been shown to induce apoptosis through the mitochondrial pathway by lowering the Bcl-2/Bax ratio and increasing the expression of cleaved caspase-3 in human non-small cell lung cancer A549 cells [37]. Therefore, we next examined the effect of MS extract on apoptosis induction.

In this investigation, we showed that the MS extract inhibits HCT116 cell proliferation through apoptosis induction. Apoptosis was characterized by typical morphological changes, including cell shrinkage, DNA condensation, fragmentation, and the formation of apoptotic bodies [17]. Our results showed that MS extract induced nuclear condensation, apoptotic bodies, and decreased *ΔΨ*m, indicators of apoptosis, as determined by Hoechst 33342 and JC-1 staining. Additionally, flow cytometric analysis confirmed apoptosis in HCT116 cells by revealing an increased sub-G1 population. These characteristics, along with specific molecular events, are associated with the activation of apoptosis signaling pathways.

We investigated the expression of apoptosis-related proteins to understand the molecular mechanisms through which MS extract triggers apoptosis. The primary regulators of the intrinsic apoptotic process are Bcl-2 family proteins, which play a role in the disruption of *ΔΨ*m [18]. When mitochondrial integrity is compromised, pro-apoptotic proteins such as cytochrome c are released, triggering the activation of caspase proteins. This suggests that MS extract induces apoptosis in HCT116 cells via the intrinsic apoptosis pathway. Caspase-8, activated in the extrinsic pathway, can cleave and activate Bid, a pro-apoptotic protein in the Bcl-2 family. This process leads to mitochondrial outer membrane permeabilization (MOMP) and the release of cytochrome c, thereby connecting the extrinsic pathway to the intrinsic pathway. Additionally, factors released from the mitochondria, such as cytochrome c, can further enhance caspase-8 activity, thereby amplifying the extrinsic apoptotic signal [16,17,18,38]. Interestingly, MS extract increased the protein expression of cleaved caspase-8 (activation) and tBid. These findings indicate potential crosstalk between the intrinsic and extrinsic pathways [16,39]. DNA fragmentation, a key indicator of apoptosis, was evidenced by the cleavage of PARP [40]. The loss of PARP activity prevents DNA repair, allowing endonucleases like CAD (caspase-activated DNase) to fragment DNA [41]. In summary, our findings demonstrate that MS extract induces apoptosis in HCT116 cells through both intrinsic and extrinsic pathways.

Similar results have been reported for mammeasin A and surangin B, compounds containing a geranyl group. These compounds, isolated from the methanol extract of *M. siamensis* flowers, exhibited strong activity against HSC-4 cells, involving apoptotic cell death [5]. Additionally, Kayeassamin A (KA), isolated from *M. siamensis* flowers, induces apoptosis in HL-60 human leukemia cells, as evidenced by chromatin condensation, DNA fragmentation, and an increase in sub-G1 phase DNA content. KA also strongly activates caspase-3 and -8, weakly activates caspase-9, and triggers PARP cleavage [12,32].

V-ATPase plays a key role in regulating intracellular pH, supporting cancer cell survival, and influencing the tumor microenvironment (TME) [24]. In cancer cells, elevated glycolysis results in excessive proton production [42]. V-ATPase facilitates the export of these protons, preventing cytoplasmic acidification, allowing cancer cells to survive in acidic conditions. Additionally, by actively transporting protons out of the cell, V-ATPase contributes to the acidification of the extracellular space, creating an acidic TME [24,43]. This acidic environment can read to promoting tumor progression and drug resistance [42,44]. Due to its critical involvement in cancer progression, V-ATPase is an attractive target for therapeutic strategies. Specifically, V-ATPases represent a promising target for cancer treatment due to their minimal impact on normal cells. Inhibiting V-ATPase activity has been associated with growth inhibition and apoptosis induction [22] through multiple mechanisms, including lysosomal dysfunction, mitochondrial damage, ROS generation, ER stress, and the inhibition of survival pathways like PI3K/Akt/mTOR and β-catenin signaling [44,45]. In our study, we found that MS extract inhibited V-ATPase activity, similar to bafilomycin A1, a well-known potent V-ATPase inhibitor. Previous research demonstrated that bafilomycin A1 induces apoptosis in pediatric B-cell acute lymphoblastic leukemia by targeting mitochondria and activating a caspase-independent pathway. The anti-cancer effects of bafilomycin A1 are linked to its inhibition of V-ATPase activity [46]. Furthermore, a study on *C. cochinchinense* extracts found that they inhibited V-ATPase activity and induced apoptosis in several cancer cell types, including MDA-MB-231, A431, and A375 cells [47]. Therefore, MS extract could be further developed as an anti-cancer drug with V-ATPase inhibitory properties. However, the detailed mechanisms by which V-ATPase inhibition induces apoptosis in HCT116 cells affected by the MS extract require further investigation. In addition, inhibiting V-ATPase directly impacts the mTOR pathway, which is a downstream component of PI3K/Akt signaling. V-ATPase helps the cell sense amino acid availability, and, when sufficient amino acids are present, it activates mTORC1 to promote cell growth [44]. It has been reported that the inactivation of mTORC1 leads to the inhibition of NF-κB signaling [48], which suggests that V-ATPase inhibition may indirectly affect NF-κB activity, potentially suppressing cell growth. Furthermore, studies have shown that the overexpression of V-ATPase B2 results in the inhibition of the MAPK pathway [49]. In the Wnt/β-catenin signaling pathway, V-ATPase is essential for maintaining the integrity of the Wnt receptor complex and regulating the degradation of components within the β-catenin destruction complex [44]. The direct inhibition of V-ATPase disrupts this process, reducing Wnt receptor signaling and promoting increased β-catenin degradation. Notably, the inhibition of V-ATPase activity by bafilomycin also blocks the Wnt/β-catenin pathway [50]. Therefore, MS extract inhibits V-ATPase, may potentially downregulate PI3K/Akt signaling, leading to reduced NF-κB activity, MAPK activation, the inhibition of the Wnt/β-catenin pathway, and enhanced apoptotic signaling. Consequently, we next investigated the effect of MS extract on these pathways.

The Wnt/β-catenin pathway regulates various cellular functions, including proliferation, differentiation, migration, and apoptosis. It suppresses apoptosis by blocking the release of cytochrome c. In most colorectal cancer (CRC) patients, the Wnt/β-catenin signaling pathway is abnormally activated. This pathway is typically initiated by the binding of TCF/LEF transcription factors with β-catenin, which activates downstream target genes, such as c-Myc and other proto-oncogenes. This promotes cell proliferation and supports tumor development and progression [51]. Consequently, downregulating the Wnt/β-catenin pathway is associated with inhibited tumor cell growth in CRC cell lines [52]. Our data support these findings, suggesting that the MS extract inhibits the growth of HCT116 cells, likely through the downregulation of β-catenin and its downstream targets. A previous study demonstrated that apigenin, a widely known flavonoid, blocked the nuclear translocation of β-catenin, thereby decreasing the expression of Wnt pathway target genes and preventing colorectal cancer cells from proliferating, migrating, invading, and forming organoids [53]. Additionally, the MS extract-treated cells showed a reduction in GSK3β, which is the first known substrate of Akt [54]. Akt phosphorylates GSK-3β at Ser9, leading to its inhibition. Since GSK-3β normally degrades β-catenin, this results in β-catenin stabilization and increased Wnt signaling [55].

Our results also indicate that MS extract decreased the expression of PI3K, p-PDK1, p-Akt (Ser473), and p-Akt (Thr308), suggesting that the PI3K/Akt pathway was inhibited. The PI3K pathway and its downstream effector Akt are key survival proteins and major contributors to chemoresistance in cancer therapy across various tumor types. When Akt is activated, it stimulates PAK1, which then phosphorylates Bad, leading to Bad’s release from the mitochondrial membrane into the cytoplasm. At the same time, Akt inhibits the translocation of Bax from the cytoplasm to the mitochondria. As a result, the overactivation of Akt promotes cell survival by enhancing Bcl-2 activity and inhibiting Bax, both of which play crucial roles in cancer cell resistance [56].

Our mechanistic study highlights the anti-cancer properties of vitexin, a flavonoid compound derived from natural sources. It has been reported that vitexin inhibits cell growth and promotes cell apoptosis by inducing G2/M cell cycle arrest, primarily through the suppression of the Akt/mTOR pathway in human glioblastoma cells [57]. Similarly, in human non-small cell lung cancer A549 cells, vitexin induces apoptosis by inhibiting the PI3K/Akt/mTOR pathway [37], which supports the findings of this study. Additionally, numerous protein targets that regulate cell motility, proliferation, and survival are phosphorylated by Akt. According to a previous study, Akt also controls NF-κB transcriptional activity [58]. Akt activation can phosphorylate and inhibit IκB (an inhibitor of NF-κB), leading to NF-κB activation and the transcription of survival and inflammatory genes [47,59]. Our findings show that MS extract reduced the expression of NF-κB, a transcription factor known for its role in preventing apoptosis. A prior study demonstrated that NF-κB inhibition promotes cell apoptosis [60]. Flavonoids, including quercetin, are recognized as inhibitors of NF-κB signaling, with evidence showing that quercetin suppresses NF-κB activity by inhibiting IκBα and p65 phosphorylation, thereby blocking NF-κB nuclear translocation [61]. Moreover, previous research has shown that the dietary phytochemical quercetin causes apoptosis in human cervical carcinoma (HeLa) cells via a mechanism dependent on the suppression of p53 and NF-κB [62]. Moreover, Akt directly phosphorylates and inhibits Raf-1, reducing MAPK activation [63]. Therefore, MAPK pathways were examined.

MAPK pathways control a range of biological functions through various cellular mechanisms. Depending on the type of cell and the specific stimulation, MAPK can act as either activators or inhibitors of these processes, including apoptosis [64]. In hepatocellular carcinoma cell lines, vitexin exerted cytotoxic effects in vitro by inducing apoptosis through the activation of the JNK MAPK pathway, as evidenced by the upregulation of p-JNK levels. The apoptosis induced by vitexin was reduced by co-treatment with the JNK inhibitor SP60012 [65]. Additionally, anthocyanins, a type of flavonoid, induce apoptotic cell death in HCT-116 cells through p38-MAPK activation [66]. These observations suggest that apoptosis induction is linked to the activation of both p38-MAPK and JNK-MAPK pathways. In our study, we demonstrated that MS extract enhanced the expression of p-p38, p-c-Jun (downstream targets of the JNK cascade), and p-ERK1/2. While the activation of p38 and JNK is typically associated with pro-apoptotic effects, ERK activation is generally linked to anti-apoptotic effects. However, there are instances where ERK1/2 signaling can also promote apoptosis [64]. ERK activity can stimulate caspase-8 activation or mitochondrial cytochrome c release, which can enhance both intrinsic and extrinsic apoptotic pathways [67]. For example, morusin, a naturally occurring prenylated flavonoid, exhibited pro-apoptotic effects on A549 and NCI-H292 cells by activating both the JNK and ERK pathways, as evidenced by changes in the ratio of phosphorylated to total protein levels. The use of a MEK/ERK inhibitor (U0126) and a JNK inhibitor (SP600125) confirmed the involvement of these pathways in apoptosis induced by morusin [68].

Taken together, our results suggest that MS extract possesses anti-cancer activity and could potentially be developed as an anti-cancer agent that acts through apoptosis induction and modulates several pathways, which has a mechanism of action similar to chemotherapy drugs, for example, 5-Fluorouracil (5-FU), a standard chemotherapeutic agent for colon cancer. 5-FU induces apoptosis by interfering with DNA synthesis and repair mechanisms. It has been shown to activate both intrinsic and extrinsic apoptotic pathways, leading to cancer cell death [69,70]. Additionally, several plant-derived compounds and experimental drugs are being investigated for their apoptotic effects in colon cancer. For instance, curcumin derived from *Curcuma longa* L. (turmeric) has been found to selectively induce apoptosis in CRC through multiple target molecules and associated signaling pathways. For example, inducing mitochondrial apoptosis via p53 activation, ROS production, inhibits NF-κB and downregulates transcription factor β-catenin [71]. Resveratrol derived from grapes modulates the Bcl-2/Bax ratio and enhances caspase activation in colon cancer [72]. Clinical trials have investigated curcumin’s efficacy in cancer prevention and treatment focusing on its apoptosis-inducing mechanisms. For instance, in colorectal cancer patients treated with curcumin, the results showed an increase in body weight, a reduction in serum TNF-alpha levels, a rise in apoptotic tumor cells, enhanced expression of the p53 molecule in tumor tissue, and a modulation of the tumor cell apoptotic pathway [73]. The studies mentioned above may help to see the effectiveness of MS extract more clearly and its potential as a treatment.

However, a limitation of this study is that it was performed only in vitro using HCT116 cells. Therefore, further studies are required in different colon cancer cell lines, as well as in vivo or using an animal model, to investigate the signaling pathways involved in its effects and to confirm these findings in a physiological context for the development of an anti-colon cancer drug. Moreover, future research should also focus on evaluating the anti-cancer activity of purifying the active compounds from the MS extract, which could pave the way for their potential use as therapeutic agents for colon cancer.

## 4. Materials and Methods

### 4.1. Materials

The reagents used in this study include MTT (3-(4,5-dimethylthiazol-2-yl)-2,5-diphenyltetrazolium bromide) and acridine orange dye, both purchased from Sigma-Aldrich (St. Louis, MO, USA). Hoechst 33342 dye [2′-(4-Ethoxyphenyl)-6-(4-methyl-1-piperazinyl)-1H,3′H-2,5′-bibenzimidazole] was obtained from Thermo Fisher Scientific (Invitrogen™, Thermo Fisher Scientific Inc., Waltham, MA, USA). Fetal bovine serum (FBS), trypsin-EDTA, Roswell Park Memorial Institute (RPMI 1640) medium, and penicillin–streptomycin were acquired from HiMedia Laboratories (Mumbai, India), while JC-1 dye (5,5′,6,6′-Tetrachloro-1,1′,3,3-tetraethylbenzimidazolylcarbocyanine iodide) was sourced from Sigma-Aldrich (Merck KGaA, Darmstadt, Germany). Lastly, the Immobilon™ Western Chemiluminescent HRP Substrate and Guava Cell Cycle^®^ reagent were purchased from Merck Millipore (Merck Millipore Corp., Darmstadt, Germany).

### 4.2. Plant Extraction

The dried flowers of *Mammea siamensis* were purchased from the Thai Lanna Herbal Industry, Chiang Mai province, Thailand, in September 2018. The extraction method used was as described in our previous study [74]. Briefly, the powder was macerated in methanol solvents at a plant sample 50 g/500 mL solvent ratio (1:10 *w*/*v*) for 3 days. The extractant was filtered using a filter cloth and Whatman^®^ Qualitative filter paper No. 4 after three days, and it was then evaporated using a rotary vacuum evaporator. The MS extract was concentrated using a vacuum desiccator for a day, resulting in 9.94 g (19.88%) of extract, which was subsequently stored at −20 °C for use in experiments. The DMSO was used to dissolve the MS extract.

### 4.3. ^1^H NMR Analysis

Proton nuclear magnetic resonance (^1^H NMR) and gradient correlation spectroscopy (gCOSY) were recorded on a 500 MHz Bruker Avance NMR spectrometer (Bruker BioSpin, Fällanden, Switzerland) in DMSO-*d6* as the solvent and referenced to the solvent peak at 2.50 ppm.

### 4.4. Cell Culture

The human colon cancer HCT116 cell line was obtained from the American Type Culture Collection (ATCC, Manassas, VA, USA). Monolayer cells were cultured in RPMI 1640 media supplemented with 10% fetal bovine serum (FBS), 100 U/mL of penicillin, and 100 µg/mL of streptomycin. A CO_2_ incubator was used to cultivate the cells at 37 °C, 5% CO_2_, and 95% relative humidity. Trypsin-EDTA at a concentration of 0.25% was used for subtraction.

### 4.5. Cytotoxicity Assay

The effect of MS extract on the cytotoxicity was determined by MTT assay. HCT116 cells were plated overnight before being exposed to different concentrations of MS extract (30, 50, 100, and 150 µg/mL) for 24 h, while 0.3% DMSO was administered to the control. Following a 2 h incubation with MTT solution (0.5 mg/mL), the formazan crystal was dissolved using DMSO. Colored formazan product was measured at 570 nm by using a Multiskan Sky microplate reader (Thermo Fisher Scientific, Waltham, MA, USA). The results were presented as percentages of cell viability, and GraphPad Prism 9 (GraphPad Prism Software, Inc., San Diego, CA, USA) was used to calculate the IC_50_.

### 4.6. Hoechst33342 Staining

Nuclear morphological changes were observed by Hoechst 33342 staining. HCT116 cells were treated with various concentrations of MS extract (30, 50, 100, and 150 µg/mL) for 24 h, while 0.3% DMSO was administered to the control group. Next, the cells were treated with Hoechst 33342 dye and allowed to incubate for 30 min. Following staining, cells were observed using a fluorescence microscope (DP73+IX71 Olympus, Tokyo, Japan).

### 4.7. JC-1 Staining

Mitochondrial membrane potential (*ΔΨ*m) was examined by JC-1 staining. HCT116 cells were treated with various concentrations of MS extract (30, 50, 100, and 150 µg/mL) for 6 h, while 0.3% DMSO was administered to the control group. After staining with JC-1 dye, the cells were photographed with a fluorescence microscope (DP73+IX71 Olympus, Tokyo, Japan).

### 4.8. Cell Cycle Analysis

To investigate cell populations in different phases of the cell cycle, we used flow cytometry. After 24 h of treatment, the cells were harvested and washed with PBS, then fixed by ice-cold 70% ethanol. Following that, the cells were stained with Guava Cell Cycle^®^ reagent. Subsequently, the DNA content was analyzed using the Guava EasyCyte^TM^ flow cytometer and GuavaSoft^TM^ software version 3.2 (Merck Millipore Corp., Merck KGaA, Darmstadt, Germany).

### 4.9. Acridine Orange (AO) Staining

To determine the effect of MS extract on V-ATPase activity, we performed AO staining. HCT116 cells were plated in 24-well culture plates and grown overnight. The following day, the cells were treated with MS extract at various concentrations (30, 50, 100, and 150 µg/mL) or with 50 nM of bafilomycin A1 (BMA), a V-ATPase inhibitor, and 0.3% DMSO was administered to the control group for 6 h. After treatment, the cells were stained with AO fluorescence dye (1 µg/mL), followed by washing with PBS, and were then observed under a fluorescence microscope (DP73+IX71, Olympus, Tokyo, Japan).

### 4.10. Western Blot Analysis

The Western blot examination revealed the protein expression findings described in our previous study [56]. Briefly, the protein extracted was separated using SDS-PAGE and subsequently transferred to PVDF membranes. These membranes were blocked with 5% BSA and incubated overnight with the primary antibody, followed by a secondary antibody conjugated with HRP (Cell Signaling Technology, Beverly, MA, USA) for 1 h, respectively. β-actin served as the internal control (Merck Millipore Corp., Darmstadt, Germany).

### 4.11. Statistical Analysis

Statistical analysis was examined by one-way analysis variance (ANOVA), followed by Tukey’s post hoc test, using SPSS software version 20.0 (IBM Crop., Albany, NY, USA). The statistical significance was accepted at *p* < 0.05 and *p* < 0.01. All the data are shown as mean ± standard deviation (SD).

## 5. Conclusions

In conclusion, this study suggests that the methanol extract of *Mammea siamensis* (MS) can inhibit cancer cell growth in HCT116 cells by inducing apoptosis through both intrinsic and extrinsic pathways. Mechanistic analysis revealed that the MS extract activates the MAPK pathway while inhibiting the PI3K/Akt, NF-κB, and GSK-3β/β-catenin pathways. Notably, MS extract was identified as a potent V-ATPase inhibitor. Based on these findings, MS extract demonstrates effective anti-cancer activity, suggesting its potential as a natural alternative for the development of colorectal cancer therapy in the future. However, to clarify the interactions between these signaling pathways affected by MS extracts, further studies, such as using siRNA, pharmacological inhibitors, and inducers, are required.

## Data Availability

The datasets analyzed during the current study are available from the corresponding author upon reasonable request.

## Supplementary Materials

The following supporting information can be downloaded at https://www.mdpi.com/article/10.3390/ph18040441/s1, Figure S1: Effect of MS extract on cell viability in Vero cells by MTT assay.
