# Supplementary material for: Mammea siamensis Flower Extract-Induced Cell Death Apoptosis in HCT116 Colon Cancer Cells via Vacuolar-Type H+-ATPase Inhibition Associated with GSK-3β/β-Catenin, PI3K/Akt/NF-κB, and MAPK Signaling Pathway
Source: Pharmaceuticals (Basel). 2025 Mar 21;18(4):441. doi: 10.3390/ph18040441 (PMC12030214; doi:10.3390/ph18040441)
Supplement: Supplementary file 1 [file pharmaceuticals-18-00441-s001.zip › pharmaceuticals-3472840-SI.pdf]

Supplementary Figures

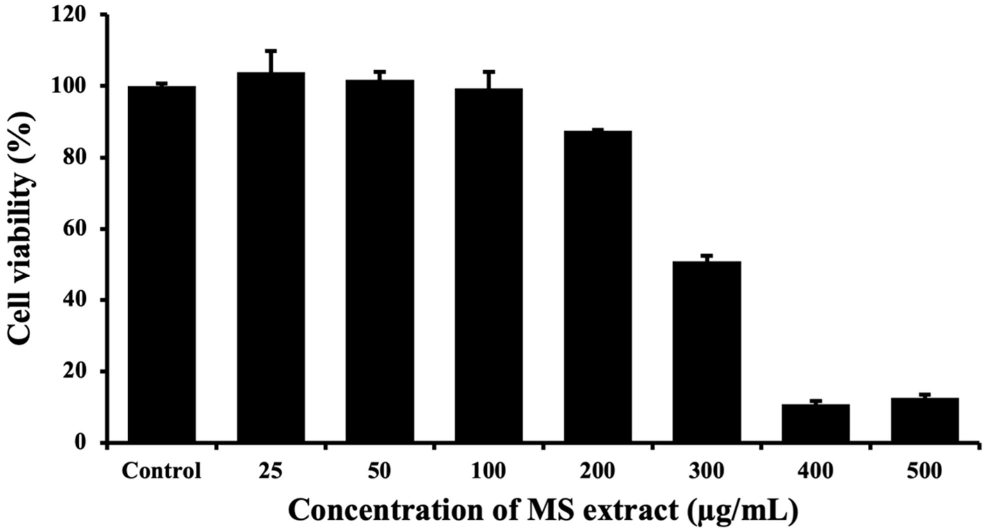

Figure S1. Effect of MS extract on cell viability in Vero cells by MTT assay. The cell lines were treated with various concentrations of MS extract for 24 h. The results were the mean values ± SD.
